# Fixing a Mismatch: The Case for Age-Aligned Kidney Allocation

**DOI:** 10.3389/ti.2025.15148

**Published:** 2025-11-07

**Authors:** Emmanouil Giorgakis, Sorabh Kapoor, Esteban Calderon, Melissa Chen, Kunal Kapoor, Alex Toledo, Chirag S. Desai

**Affiliations:** Department of Surgery, The University of North Carolina at Chapel Hill, Chapel Hill, NC, United States

**Keywords:** kidney transplant allocation, kidney donor profile index, estimated post transplant survival score, older donors, age-matched allocation

## Abstract

Despite recent advances, deceased donor kidney transplant allocation in the United States does not sufficiently account for the mismatch between donor and recipient age. This misalignment often leads to a suboptimal use of scarce resources. This viewpoint calls for restructuring of current kidney allocation strategies, advocating for a more intentional, age-matched approach that prioritizes better long-term quality kidneys for proportionally younger patients and encourages the use of older donor kidneys in similarly aged recipients. Drawing on the National Scientific Registry of Transplant Recipients data, clinical observations, and ethical reasoning, we argue that incorporating age in the organ allocation algorithms may improve both equity and utility in organ distribution. We also advocate for revision of the kidney donor risk calculators and placing a cap on the pre-emptive wait-time. Such realignments may reduce organ discard rates, enhance long-term graft utility, alleviate decision-making burdens on patients, and decrease the need for re-transplants on younger patients. To achieve this, recalibrations in allocation algorithms and reframing of what constitutes a “good” kidney are required. The goal is not to limit choice, but to structure a framework that maximizes benefit across populations while maintaining fairness towards a more sustainable model of transplant care.

## Introduction

Kidney transplantation remains the optimal treatment for end-stage renal disease, yet current allocation practices may inadvertently favor less equitable distribution, particularly between older and younger patients.

Most transplant surgeons have encountered this situation: The older patient, declining a good quality organ because of a higher Kidney Donor Profile Index (KDPI) - mostly age-driven. That older kidney, which an age-matched candidate declined, may end up being used by a younger patient or get discarded. The same patient will remain on the waitlist arena to compete over the same scarce supply of -inevitably- younger organs. This older patient may not even be on dialysis yet; however, they still rank higher than younger dialysis patients who have shorter wait times: dialysis patients do not necessarily precede their pre-emptive counterparts on the allocation race; it only takes a snapshot of GFR <20 mL/min/1.73 m^2^ to qualify for enlisting. Once captured, a pre-dialysis patient may remain on the list for a decade or more, at times outflanking dialysis patients with shorter wait times.

But this is a zero-sum game: when older patients preferentially decline high-quality, age-matched grafts in favor of younger ones, it consequently results in fewer available grafts for younger patients. This leads to these questions: Should there be donor-recipient age-matching? Should KDPI weigh so heavily on organ offer acceptance? Given the recent advancements in DCD outcomes with the increasing use of normothermic regional perfusion (NRP), how relevant is the KDPI calculator for such offers? What is the impact of patients’ health literacy on organ acceptance decision-making? Should there be guardrails to protect patients from declining suitable offers and limit the discarding of usable older organs?

## Donor and Recipient Age

Post-kidney transplant (KT) survival varies significantly by recipient age. Wolfe et al, in their seminal NEJM paper on outcomes among recipients of first cadaveric transplant, using US Renal Data of 252,358 patients, showed that projected years of life without transplantation vs. with transplantation doubled across all age groups [1]. Recipients under the age of 39 have the most impressive increase in life expectancy, exceeding 20 years: on patients <19 years, life expectancy without vs. after KT was 26 vs. 39 years, respectively; on patients 20–39 years, it was 14 vs. 31 years; on patients aged 40–59, life expectancy doubled from 11 to 22 years; on patients aged 60–74, life expectancy extended from 6 to 10 years (table 1). Table 2 illustrates the age distribution of patients on the KT waitlist, based on the 2021 Scientific Registry of Transplant Recipients (SRTR) Annual Data Report, and the estimated percentage of total deceased donors per respective age group: 63.6% of waitlisted patients were over 50, with over 20% being >65. 30% of organs were from donors less than 34 [2]. 60% of organs were from donors <50 (est. KDPI ∼60%). 11% were over 65 (KDPI >85%; “high KDPI” kidneys).

**TABLE 1:** Outcome among recipients of first deceased donor renal transplant according to age at time of enlisting (1991–1997).

Age group (years)	Relative risk 18 months after KT (95%CI)	Projected years of life without KT	Projected years of life with KT
All recipients	0.33 (0.30–0.35)	10	20
0–19	0.33 (0.12–0.87)	26	39
20–39	0.24 (0.2–0.29)	14	31
40–59	0.33 (0.29–0.37)	11	22
60–74	0.39 (0.33–0.47)	6	10

**TABLE 2:** Age distribution of waitlisted kidney failure patients and of deceased kidney donors (OPTN/SRTR, 2021).

Age group (years)	% Of waitlisted patients	Estimated % of total deceased donors
0–17	1.6	7
18–34	11.4	23
35–49	23.4	30
50–64	42.5	33
≥65	21.1	11

## KDPI: Time to Revisit?

**FIGURE 1:**
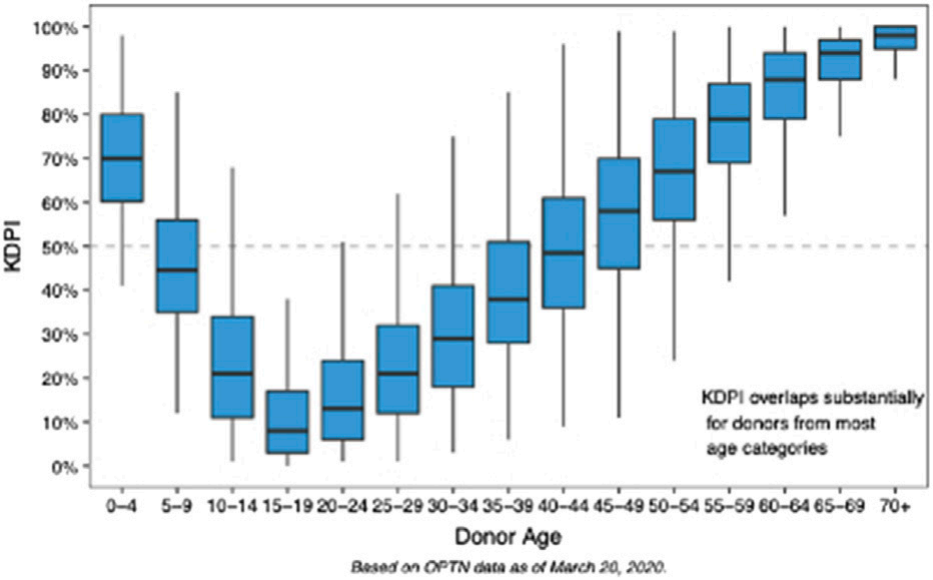
Distribution of Kidney Donor Profile Index (KDPI) by Donor Age. Box-and-whisker plots demonstrate the variation in KDPI across donor age groups (OPTN data). KDPI increases progressively with donor age, with significant overlap across age strata, particularly in mid-to older-age groups. Younger donors (age 85%). These data highlight the age-dependent nature of KDPI.

The KDPI, implemented in 2014, aimed to enhance kidney allocation by quantifying donor organ quality [3, 4]. The KDPI is derived from the Kidney Donor Risk Index (KDRI). KDRI is calculated using these donor characteristics: age, height, weight, history of hypertension, diabetes, cause of death, serum creatinine, and DCD status [3]. Donor age is heavily weighted in the KDPI model, with donors >60 years of age conferring organs with a KDPI >85% (Figure 1) [4]. Despite the intent, the KDPI application has led to discards of potentially transplantable organs [5].

**FIGURE 2:**
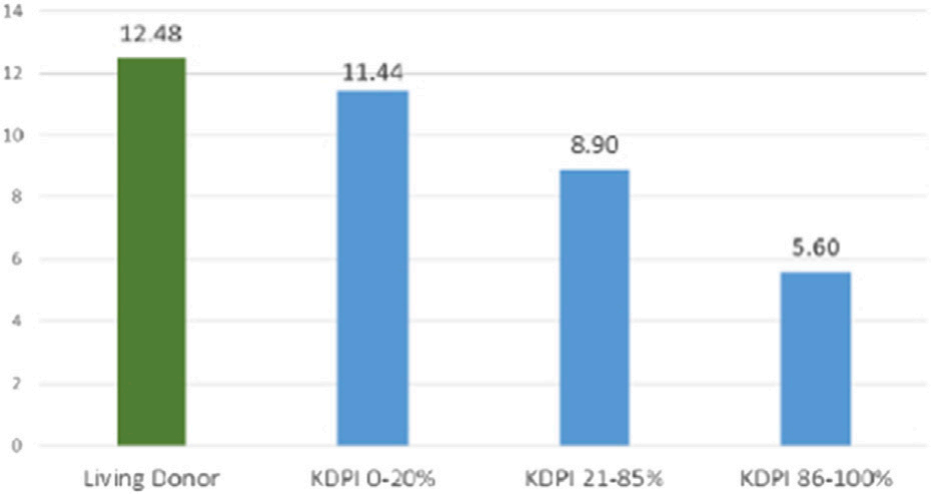
Graft Half-Life by Donor Type and KDPI Category. The bar graph delineates the observed graft half-life in years for kidneys from living donors and deceased donors, stratified by KDPI group (OPTN data. Living donor grafts exhibit the longest median survival (12.5 years), which approximates the half-life of low KDPI (0–20%) deceased donor kidneys (11.4 years). This is followed by moderate KDPI (21–85%) kidneys, with a median survival of 8.9 years, and high KDPI (86–100%) kidneys, with a median survival of 5.6 years.

**FIGURE 3:**
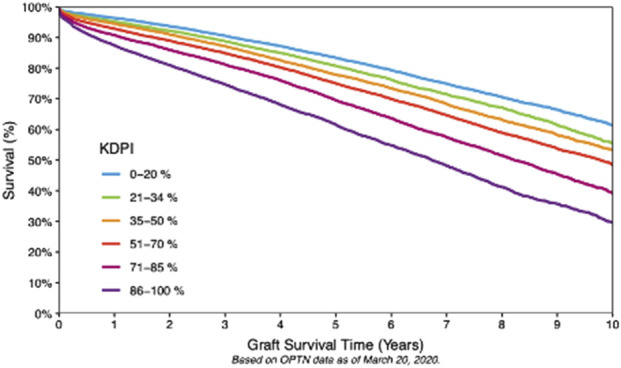
Ten-Year Graft Survival According to KDPI. Ten-year Kaplan–Meier graft survival rates, stratified according to KDPI categories (OPTN data). Low KDPI (<20%) kidneys exhibit the highest long-term survival rates. The graph also underscores the often-underestimated advantages of employing higher KDPI kidneys in appropriately matched recipients, taking into account their projected health-adjusted life expectancy (HALE) post-transplant compared to their projected years of life lost (YLL) and years lived with disability (YLD) in the absence of transplantation.

While low KDPI kidneys (<20%) are discarded <10% of the time, discard rates for higher KDPI kidneys can exceed 50% due to concerns about inferior outcomes. Nonetheless, these organs may still offer years off dialysis and get patients transplanted while they are still fit for transplant [5, 6]. Figure 2 illustrates the inverse relationship between KDPI scores and graft survival. A deceased donor kidney with KDPI <20% has an estimated half-life of 11 ½ years after transplant. Approximately 24.9% donors have a KDPI <20% [5]. Such organs are prioritized to younger (EPTS <20%) patients, thus reducing the need for retransplant, a leading cause of needing a KT. 65% of deceased donor kidneys have KDPI 21%–85%, with an average ½ life of 9 years (Figure 2) [2–5]. Kidneys with KDPI exceeding 85% have a ½ life of 5 ½ years. Ten-year graft survival was higher (>60%) at the KDPI <20% group and lower (30%) at the KDPI >85% group (Figure 3).

Recent US and European studies have shown that NRP significantly reduces the incidence of delayed graft function (DGF) while also decreasing the risk of discard [7]. Multiple registry and multicenter studies report DGF rates of 13%–25% for NRP versus 27%–35% for standard DCD recovery, along with a lower risk of 1-year graft loss and improved early renal function [7–12]. NRP also increases the proportion of DCD kidneys that are ultimately transplanted, with utilization rates approaching those of donation after brain death [11, 12]. Although NRP is not a uniformly applied DCD procurement practice, the proportion of NRP DCD kidney allografts has been increasing, favorably impacting DCD kidney utilization and outcomes. It would thus be reasonable to consider adjusting the KDPI formula to account for the mitigating effect of NRP (if used), accurately reflecting the predicted graft quality and avoiding unnecessary organ declines or discards.

## The Estimated Post-Transplant Survival (EPTS) Score

To incorporate post-transplant life expectancy into the allocation algorithm, the United Network for Organ Sharing (UNOS) has introduced the EPTS score. As the name implies, EPTS is a numerical tool to predict how long a KT candidate is expected to survive following transplantation [13]. It ranges from zero to 100%, with lower scores indicating candidates are expected to have more prolonged post-transplant survival. The score is calculated using four recipient factors: age, time on dialysis, history of diabetes, and prior organ transplants [8].

EPTS score’s purpose is to better match donor organ longevity with recipient survival potential, thereby improving long-term transplant outcomes and optimizing the use of high-quality donor kidneys. Alas, the EPTS score is not perfect in accurately projecting post-transplant survival. EPTS may inadvertently harm young patients with failed prior transplants and already on dialysis, a not too uncommon scenario. Assume the example of a 27-year-old type-I diabetic, after two failed KTs, 8 years on dialysis. This patient would score the same (36%) as a 64-year-old pre-emptive candidate enlisted for a first transplant [13]. These two candidates may compete for the same younger donor allografts. The older, pre-emptive patient may even outrank the 27-year-old if enlisted for a more extended period. However, the younger candidate would have more projected years of life lost (YLL) and more years living with disability (YLD) without a KT and, conversely, more health-adjusted life years (HALE) ahead of them following a KT, compared to the older counterpart [14]. This paradigm underscores the flawed nature of a metric central to allocation.

## The Case for Age-Matched Organ Distribution

Per UNOS, the estimated ten-year patient survival for EPTS scores zero-20%, 21%–80%, and 81%–100% were ∼85%, 50%–70%, and ∼35%, respectively (Table 3). Candidates with an EPTS score of 0%–20% are considered to have the best expected outcomes. They are indeed prioritized to receive kidneys from donors with KDPI <20%. However, there is no similar prioritization tool for “high EPTS” candidates and higher KDPI organs. A kidney from a 70-year-old DCD donor with a terminal creatinine of 0.9 mg/dL and hypertension (KDPI 96%) may still be the best option for a 75-year-old patient who has been on dialysis for 4 years, already had a previous transplant (EPTS 95%), and will soon no longer be fit for retransplant. A 68-year-old diabetic with 4 years on dialysis (EPTS 94%) would be a good match for a kidney from a 65-year-old hypertensive brain-dead donor with a terminal creatinine of 1.5 mg/dL (KDPI 88%). By receiving a timely transplant with a higher KDPI kidney (projected ½ life 5–8 years), these elderly patients can be taken off dialysis, freeing up longer-lasting organs for younger candidates and thus reducing their need for retransplant.

**TABLE 3:** 10-year EPTS survival estimates (2012–2022).

EPTS group	Estimated 10-year survival (%)
0%–20%	∼75%
21%–40%	∼70%
41%–60%	∼63%
61%–80%	∼55%
81%–100%	∼45%

## Placing a Cap on Pre-emptive Time

The purpose of enlisting pre-emptive patients is to keep them away from dialysis, which is known to be associated with higher morbidity and mortality risk [14, 15]. Indeed, ideally, we would want to be transplanting everyone while they are pre-emptive, eliminating the need for dialysis. Yet, this is far from the present reality. There are not enough organs to meet the need. Liberal, prolonged, pre-emptive enlisting can distort fair allocation, limiting transplant access for someone with a shorter wait-time yet already on dialysis. Consider a 40-year-old who has been on dialysis and on disability for 5 years, outscored by a 68-year-old pre-emptive patient on the race for a KDPI 35% graft, due to the latter’s longer captured wait-time. A more effective approach would be to place an upper limit on the preemptive time to prevent scenarios where a preemptive patient has significant leverage.

## Success Stories From Other Allocation Systems

Placing donor-recipient age guardrails would prioritize the use of younger grafts to serve patients of similar age groups better. This approach would a. increase the relative supply of younger organs to younger people with longer projected years of healthy life, thereby maximizing graft utility and societal beneficence, and b. Put older grafts to good use—grafts that would otherwise be at risk of being discarded.

Other transplant systems have demonstrated the ability to serve the needs of patients across the age spectrum more fairly. In 1999, Eurotransplant established the Eurotransplant Senior Program (ESP) to match the functional capacity of organs from donors ≥65 years old with the needs of recipients aged ≥65^16^. These organs were allocated within a narrow geographic area. In 5 years (1999–2004), the availability of elderly donor allografts doubled, while the wait-time of the ESP patients dropped, without negatively affecting graft and patient survival. ESP led to shorter cold ischemic time (CIT) and less DGF of older donor allografts, showcasing an effective organ allocation system from elderly donors [16, 17].

The French Transplant System, primarily managed and coordinated by the Agence de la Biomédecine implemented in 2015 the Unified Allocation Score (UAS), to guide decision-making [18]. UAS score includes, among other factors, dialysis duration, recipient age, and donor-recipient age difference. French system age-matching principles are to avoid allocating young donor kidneys to significantly older patients (>5 years older), and prevent older kidneys from being assigned to much younger recipients (>20 years) [18, 19]. The UAS implementation has led to better access to transplant for the younger patients and more rapid access for the older ones [19].

In the US, the latest OPTN liver allocation policy change, along with a rise in organ demand for high-quality organs for alcoholic hepatitis patients, placed a natural selection pressure on the transplant networks to either pursue local DCD livers that were previously discarded or perish [20, 21]. After an adjustment period, this Darwinian challenge led to an unprecedented rise in DCD utilization—albeit catalyzed by advanced preservation technologies—and a net rise in liver transplant rates [20–23]. Taking this as an example, prioritizing younger and better-quality kidneys for younger candidates (with longer healthy life expectancies), while expediting the allocation of older grafts to local older age candidates, would minimize the discard and DGF risks due to protracted CIT. Prioritizing local allocation of older grafts to older recipients would potentially increase overall transplant rates and shorten waitlist times for all age groups.

## Out-Of-Sequence Allocation

Proper out-of-sequence organ utilization is essential to maximize graft utilization and salvage transplantable grafts-at-risk, ultimately resulting in shorter waiting times and fewer organ discards [24–27]. As such, if appropriately used, it makes moral sense [28, 29]. Placing policy safeguards, e.g., by prioritizing older kidneys to older recipients residing locally, would convert an out-of-sequence allocation to an “in-sequence”, making what most surgeons would rather do: expeditiously transplant older kidneys locally, rather than wasting hours awaiting the allocation algorithms to run down the list only to be ultimately declined from remote centers; a delay often leading to organ discard or higher DGF if transplanted.

Introducing a “high KDPI” donor to “high EPTS” recipient concept (e.g., KDPI >70% or donor aged >59 years prioritized to EPTS >60%, Table 3; Figures 1, 3) or implementing local expedited allocation of older grafts similar to the ESP model, would allow for an “in-sequence” allocation of these grafts-at-risk. Reversely, prioritizing the younger grafts (e.g., from donors aged <50 years or with KDPI <60–70%) to proportionally younger recipients would potentially trigger increased utilization of local older donor resources to cover the needs of higher KDPI (older) candidates, potentially reaching a critical threshold akin to the observed changes in DCD liver allocation noted in the early 2020s-which led to the unprecedent rise in DCD liver graft utilization in the US over the most recent years [20, 21].

Of note, the authors do not advocate using age as the predominant or sole determinant of kidney allocation. Ultimately, the decision to use or decline is incumbent upon the surgeon’s determination of whether the organ is suitable for the intended recipient or whether it may not be ideal for the top-ranking patient, yet still a good fit on a different recipient, taking into account all other clinical, laboratory, and histopathological parameters. Non-usable grafts based on functional parameters and histopathological characteristics will remain non-usable, irrespective of donor or recipient age. However, prioritizing the initial organ offer of an older graft to, e.g., a locally residing older recipient so that the graft may be implanted expeditiously, may render an organ-at-risk (of discard) a wisely utilized, life-saving graft.

## Health Literacy and the Role of the Transplant Provider as Patient Advocate and Custodian of Organ Transplantation Tenets

Younger donor grafts have more prolonged survival. However, given that both donor and waitlisted population age distributions are increasingly skewed to the right, it would be impossible to generate enough young donor allografts to accommodate everyone. On the other hand, older recipients have inferior long-term outcomes even if they get the top-quality organs, due to their inherent comorbidities and higher risk of post-transplant infection-related mortality [30]. It would be fair to expect older recipients to be content receiving transplantable organs from older donors, sparing the rest for recipients with life expectancy better matched to the graft’s survival. However, passing the burden of a socially conscious decision on the recipient at the time of organ offer is unjust [31]. Also, patients’ healthcare literacy often influences their decision-making [31]. Some may overestimate their understanding of medical risks and graft quality or have unrealistic expectations [31]. The complexity of multidisciplinary transplant teams may also overwhelm patients, sometimes creating conflicting impressions. Clear, coordinated communication and decision support tools are essential to bridge this gap. The SRTR has introduced an online Kidney Transplant Decision Aid, to help patients make an informed decision [32, 33]. However, this is hardly sufficient to help them navigate through such a complex decision, often to be taken at a moment’s notice, at the time of the organ offer. Those determinations are best made at the public policy and allocation level, with the support of transplant professionals who are incentivized—and sworn by oath—to have an eye on their patients’ best interests. The onus is therefore on the transplant system to guide patients to the right decision, weighing both their interests and the public’s best interests in mind.

## Implementation

To implement any of the modifications mentioned above, a revision of the respective UNOS policies would be needed. It would be prudent to pilot these changes before broader implementation—potentially in areas or centers experiencing the longest waitlists. If such policy alterations are ever contemplated, there would inevitably be obstacles to address, primarily encompassing preconceptions among the transplant community, healthcare providers, and patients. The optimal approach would entail educating all stakeholders and providing comprehensive training of the new algorithms before widespread adoption.

## Conclusion

Our donor and recipient populations are only getting sicker and older. It is incumbent upon our transplant policymakers and stakeholders to implement organ distribution models to mitigate the waste of graft-years and unnecessary organ discards at the extremes of donor and recipient age, perhaps by following the paradigm of countries that have addressed this aspect more successfully. KDPI and its dominant role in organ allocation should be refashioned or even dethroned. The utility of the EPTS score, particularly at both extremes of its spectrum, needs to be revisited. An upper limit may need to be established on pre-emptive wait-time. Older kidneys should be prioritized locally for their waitlisted age-peers.

There is a fine line between equitable organ access and a socially responsible organ distribution. We ultimately need to find the courage to strike a balance between our egalitarian and utilitarian approaches, building an organ allocation framework that maximizes benefit across age populations while maintaining fairness and sustainability of transplant care.

## Data Availability

The original contributions presented in the study are included in the article/supplementary material, further inquiries can be directed to the corresponding author.
